# Linking Obesity with Colorectal Cancer: Epidemiology and Mechanistic Insights

**DOI:** 10.3390/cancers12061408

**Published:** 2020-05-29

**Authors:** Pengfei Ye, Yue Xi, Zhiying Huang, Pengfei Xu

**Affiliations:** 1 College of Clinical Medicine, Henan University of Science and Technology, Luoyang 471003, China; ypf99168@126.com; 2 Center for Pharmacogenetics and Department of Pharmaceutical Sciences, University of Pittsburgh, Pittsburgh, PA 15213, USA; YUX42@pitt.edu; 3 School of Pharmaceutical Sciences, Sun Yat-sen University, Guangzhou 510006, China; hzhiying@mail.sysu.edu.cn

**Keywords:** obesity, colorectal cancer, epidemiology, hormones, inflammation, gut microbiota, bile acids

## Abstract

The incidence of obesity and colorectal cancer (CRC) has risen rapidly in recent decades. More than 650 million obese and 2 billion overweight individuals are currently living in the world. CRC is the third most common cancer. Obesity is regarded as one of the key environmental risk factors for the pathogenesis of CRC. In the present review, we mainly focus on the epidemiology of obesity and CRC in the world, the United States, and China. We also summarize the molecular mechanisms linking obesity to CRC in different aspects, including nutriology, adipokines and hormones, inflammation, gut microbiota, and bile acids. The unmet medical needs for obesity-related CRC are still remarkable. Understanding the molecular basis of these associations will help develop novel therapeutic targets and approaches for the treatment of obesity-related CRC.

## 1. Introduction

Obesity is associated with various metabolic disorders [1], such as diabetes, non-alcoholic fatty liver diseases, cardiovascular diseases, hypertension, and obstructive sleep apnea syndrome, as well as with some cancers [2,3,4], including esophageal adenocarcinoma, multiple myeloma, cardia cancer, colorectal cancer (CRC), cholangiocarcinoma, pancreatic cancer, breast cancer, endometrial cancer, ovarian cancer, and renal cancer. Obesity is closely related to increased incidence and progression of these cancers, and it is estimated to cause about 20% cancer-associated deaths [5,6]. In this review, we mainly focus on the epidemiology of obesity and CRC in the world, the United States, and China, and the molecular mechanisms of obesity contributing to CRC.

## 2. The Epidemiology of Obesity and CRC

### 2.1. The Epidemiology of Obesity

**Figure 1:**
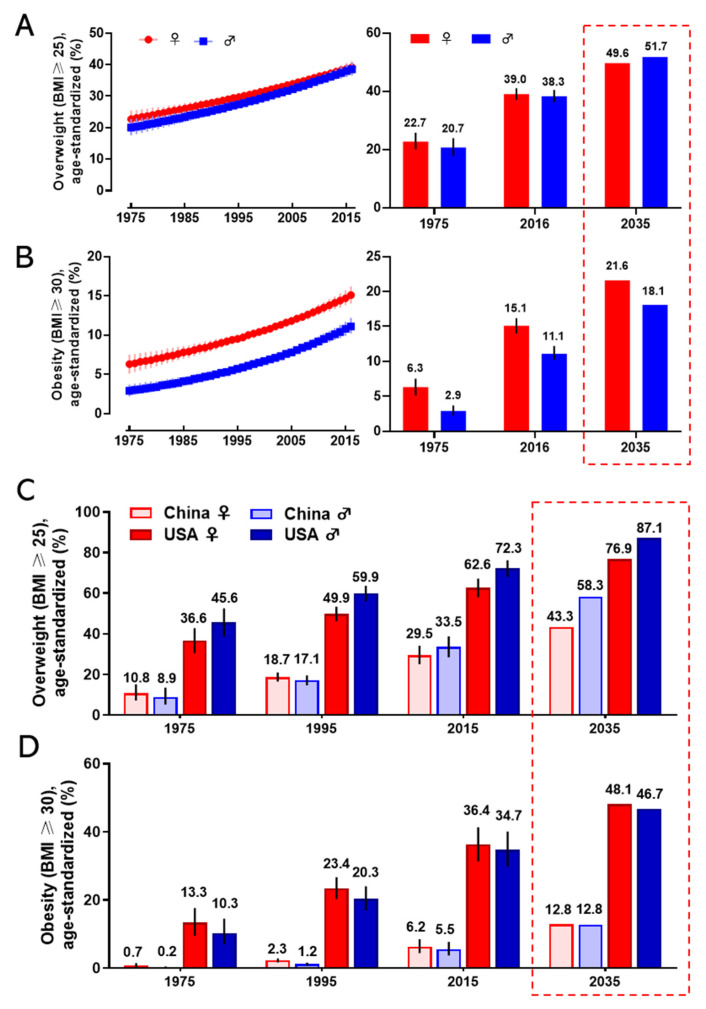
The prevalence of overweight and obesity in women and men. The global prevalence of overweight (**A**) and obesity (**B**) in women and men from 1975 to 2016 (left), and the value in 1975 and 2016, and the prediction in 2035 (right). The prevalence of overweight (**C**) and obesity (**D**) for women and men in 1975, 1995, 2015, and the prediction in 2035 in China and the United States. The predicted values were boxed with the dashed line. Data are from the WHO website.

Obesity has become a worldwide health burden. Body mass index (BMI) is a typical value derived from the weight and height to define overweight (25 ≥ BMI < 30) and obesity (BMI ≥ 30) in adult men and women. According to the World Health Organization (WHO) reports, the rate of obesity has nearly tripled globally since 1975. In 2016, about 2 billion adults were overweight, and more than 650 million of them were obese. The worldwide prevalence of overweight was 22.7% in women, and 20.7% in men in 1975; it was markedly increasing to 39.0% and 38.3% in 2016 and it will arrive at 49.6% and 51.7% in women and men respectively in 2035 (Figure 1A). The global prevalence of obesity was 6.3% for women, and 2.9% for men in 1975; this proportion rose to 15.1% (women) and 11.1% (men) in 2016 and will reach 21.6% (women) and 18.1% (men) in 2035 (Figure 1B). The regions with the highest prevalence of obesity are American and European [7]. With an estimated 89.6 million obese, China has the largest population of obese in the world [8]. Since 1975, the prevalence of overweight and obesity in men and women every two decades in China and the United States is shown in Figure 1C,D. If the current trends continue, as predicted, the prevalence of overweight and obesity in the USA will reach 76.9% (women) and 87.1% (men), and 48.1% (women) and 46.7% (men) in 2035, respectively. The prevalence of overweight and obesity in China will reach 43.3% (women) and 58.3% (men), and 12.8% both in women and men in 2035, respectively. Obesity has been a serious threat to human health and a heavy financial burden of health insurance, which affects the normal physiological function of humans.

### 2.2. The Epidemiology of Colorectal Cancer

**Figure 2:**
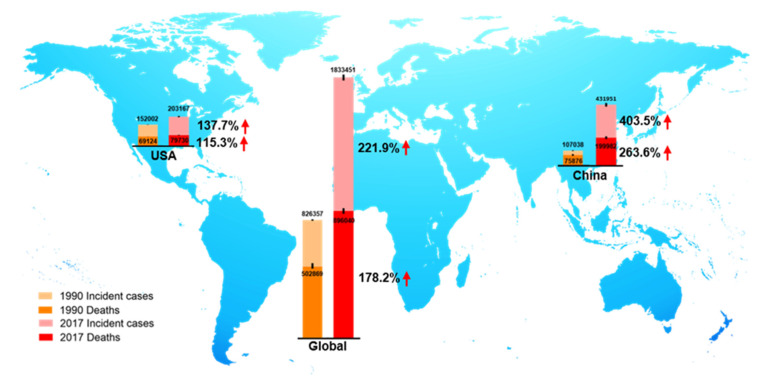
The incident cases and deaths of colorectal cancer (CRC) from 1990 to 2017 in the world, the USA, and China. Data are from Global Burden of Disease Study 2017 (GBD 2017).

CRC is the third most prevalent cancer and is also the third leading cause of cancer-associated death globally in both men and women from the 1980s [9,10]. In 2018, there were 1.8 million new CRC cases, causing 0.86 million deaths worldwide, according to global cancer statistics [11]. Currently, there are more than 1 million CRC survivors in America. Based on American Cancer Society statistics 2020, the estimated numbers of new CRC cases and deaths in the United States are approximately 150,000 and 54,000, respectively [9]. Global Burden of Disease Study 2017 (GBD 2017) reported the numbers of incident cases and deaths of CRC globally, in the USA, and China from 1990 to 2017, as shown in Figure 2. We observed that in 1990, incident cases and deaths of CRC are about 107,000 and 76,000 in China, and about 432,000 and 200,000 in 2017, respectively [12]. Over the past 27 years, the incidence cases of CRC have doubled worldwide, and been increased three times in China. The unmet medical needs of CRC have been a growing public health issue.

Growing epidemiological data indicated a strong positive correlation between obesity and colorectal carcinogenesis [13,14,15]. General obesity causes a higher risk of colon cancer in males compared to females, and it has a stronger association with colon cancer than rectal cancer in both genders [16,17]. Dose-response meta-analysis reported that body weight gain of 10 kg was accompanied by approximately 8% increased risk of CRC [18,19]. Early-life obese individuals are at greater risk of developing CRC in adulthood [13,18,20]. As expected, body weight loss by bariatric surgery reduces about 27% risk of CRC [21,22]. Understanding the association between body weight and the risk of CRC is essential to guide body weight management for CRC patients.

## 3. The Mechanistic Insights Linking Obesity with CRC

Although increasing evidence suggests the positive correlation between obesity and CRC, the underlying molecular mechanisms are still not fully understood. Obesity-induced abnormal lipid metabolism, adipokines and hormones, chronic inflammation, gut microbiota dysbiosis, and disrupted bile acid homeostasis may play important roles in the complex metabolic regulation of CRC tumorigenesis.

### 3.1. Nutriology

Obesity is excess body adiposity, especially ectopic deposition of white adipose tissues. Mature adipocytes (white adipocytes) act as an energy bank to store and release energy [23]. Systemic and local energy metabolic homeostasis is primarily controlled by adipocytes [24,25]. Tumor cell growth requires a lot of energy. Understanding whether and how tumor cells get energy directly from the adipocytes helps develop new therapeutic strategies.

**Figure 3:**
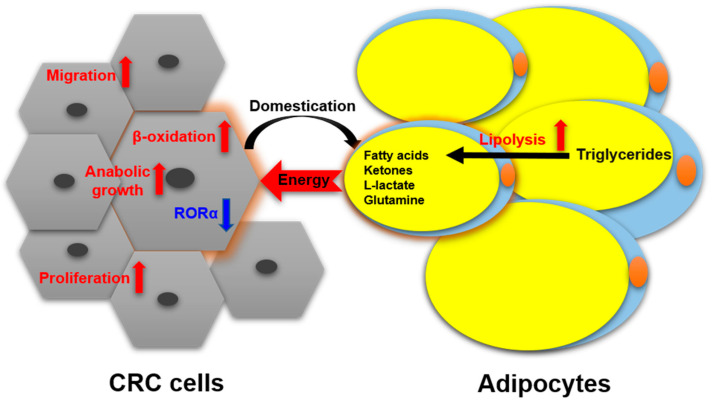
The crosstalk between CRC cells and adipocytes in nutriology.

Nieman et al. reported [26] that intra-abdominal tumors are more likely home to and proliferate in the omentum majus, which is an organ mainly composed of white adipocytes. Adipocyte-tumor cell coculture induces lipolysis in adipocytes and β-oxidation in tumor cells, resulting in the rapid proliferation of tumor cells. An emerging concept in cancer metabolism is that the adipocytes surrounding tumors provide energy or nutrients for the anabolic growth of cancer cells [27,28,29]. We validated this concept by observing more adipocytes surrounding colorectal tumor tissues than normal tissues in clinical pathological sections [30]. In in vitro experiments, we found adipocyte-conditioned medium promotes proliferation and migration of colon cancer cells (SW480 and C26) through retinoic acid-related orphan α (RORα), which is a lipid metabolism-associated nuclear receptor [30]. Sadahiro et al. reported that primary adipocytes, preadipocytes, and adipose tissues enhanced the growth of colon cancer cells (CACO-2, T84, and HT29) in the cocultured system [31]. Adipocytes are part of tumor microenvironment. It is domesticated to produce and transfer energy-rich metabolites to tumor cells, including free fatty acids, glutamine, ketones, and L-lactate, and promote the growth and migration of tumors [29]. The summarized crosstalk between CRC cells and adipocytes in nutriology is shown in Figure 3. CRC cells domesticate adipocytes which supply energy or nutrients to cancer cells for further rapid growth.

Cancer cachexia (CC), also known as wasting syndrome, is characterized by weight loss in cancer patients. It is caused by tumor factors and regulated by catabolic metabolism [32]. This complex multifactorial metabolic syndrome often accompanies increased lipolysis in adipose tissues. A total of 54% of colon cancer patients suffer from CC that causes about 20% of cancer-associated deaths [33,34]. It might be a piece of evidence that adipose tissues provide nutrients for tumor growth in systemic nutriology.

Understanding the role of adipocytes in tumor microenvironment is critical to the discovery of new strategies. Targeted blocking energy transfers might be novel therapies for the treatment of CRC.

### 3.2. Adipokines and Hormones

Adipose tissues have long been thought to be energy storage tissues as the body accumulates excess nutrients and to resist cold temperature [35]. It is currently regarded as a highly active endocrine or metabolic organ [36]. It liberates more than twenty kinds of hormones and adipokines, such as estrogens, insulin, insulin-like growth factors (IGFs), leptin, adiponectin, apelin, visfatin, resistin, chemerin, omentin, nesfatin, vaspin, inflammatory cytokines (e.g., tumor necrosis factor-alpha (TNF-α), chemokine (C-C motif) ligand 2 (CCL2), plasminogen activator inhibitor-1(PAI-1), and the interleukin families (e.g., IL-1β, IL-6, IL-8, IL-10, IL-27, and IL-31). The related adipokines and hormones and their functions in the development and progression of CRC are introduced below.

#### 3.2.1. Insulin and IGFs

The insulin/IGFs system is a major driver in the pathogenesis of CRC. This system consists of insulin, insulin receptor (IR), IGF-1 and -2, IGF-1 receptor (IGF-1R), IGF-binding protein (IGFBP)-1 and -2, and IR substrates (IRS) 1 and 2 [37]. Overweight generally increases the levels of insulin and IGF-I and decreases the levels of IGFBP-1 and IGFBP-2 in serum [38]. Insulin and IGFs have been reported to promote the proliferation of HCT116 and HT29 colon cancer cell lines through activation of the phosphoinositide 3-kinase (PI3K)/Akt signaling pathway [39,40,41]. PI3K/Akt signal pathway is an important therapeutic target for treating colon cancer [42,43]. Tyrosine-protein kinase Src is a non-receptor tyrosine kinase encoded by the *SRC* gene in humans [44]. It regulates PI3K/Akt pathway through phosphorylation of PI3K. Src also plays a critical role in the transformation and growth of CRC cells. Knockdown or inhibition of Src inhibited cell metastasis and proliferation in human cancer cells SW480 and HT29 [45,46]. Phosphorylated IR (pIR) was highly expressed in low-grade colorectal adenocarcinoma, which indicated activation of IR is an early event in CRC tumorigenesis [47]. The expression levels of IGF1 and IGF-1R were increased in colorectal carcinomas, compared with normal colonic mucosa. Overexpression and activation of IGF1-R can activate Src, leading to elevated proliferation and migration of colon cancer in vitro [48]. Renehan et al. reported that IGF-2 SD scores (SDS) were slightly increased in CRC patients compared to healthy controls, and it showed a more dramatic increase in advanced colonic carcinomas compared with earlier stages, but the scores dropped down immediately after curative resection [37]. Taken together, the insulin and IGFs system plays an important role in the pathogenesis and prognosis of CRC through independent or joint signaling networks.

#### 3.2.2. Leptin and Adiponectin

Leptin, a peptide hormone encoded by *Ob* gene, is mainly secreted by adipose tissues, which informs the brain that the energy runs out in the liver through binding to leptin receptors [49,50,51]. Obese individuals have high levels of circulating leptin, because of leptin resistance [52]. Leptin is a risk factor for CRC [53,54]. The expression of leptin is increased in human colorectal tumors and is associated with tumor progression and clinic pathological parameters [55]. Soluble leptin receptor (sOB-R) is a potential marker of leptin resistance. European Prospective Investigation into Cancer and Nutrition (EPIC) cohort also showed circulating sOB-R inversely correlated with the risk of CRC [56,57]. In azoxymethane (AOM) induced murine colon cancer model, Leptin-deficient (*ob/ob*) and leptin receptor-deficient (*db/db*) mice showed inhibited tumor growth through Wnt signaling pathway [54]. Leptin increases cell proliferation and prevents apoptosis in HT29 cells through phosphorylation of c-Jun NH2-terminal kinase (JNK). JNK phosphorylation stimulates a cascade of downstream protein phosphorylation, including Janus kinase 2 (JAK2) and PI3K/Akt, then activates signal transducer and activator of transcription (STAT3) and activator protein 1 (AP-1) [58]. Leptin promotes cell migration and lamellipodial extension in human CRC cell lines LS174T and HM7 through activation of Rho family of GTPases, including ras homolog family member A (RhoA), cell division control protein 42 (Cdc42), and ras-related C3 botulinum toxin substrate 1 (Rac1) [59]. Adipose tissues secreted leptin inhibits mitochondrial respiration rate in HCT116 cells [60,61]. Leptin provides a link between obesity and the risk of CRC, it is a sensitive marker of obesity-induced hormonal aberrations and may be directly involved in CRC tumorigenesis.

Adiponectin is a protein hormone encoded by *ADIPOQ* gene in humans [62]. It is one of the most abundant hormones released from adipose tissues and performs an essential function in obesity-associated cancers. The expression and circulating levels of adiponectin are reduced in most obese individuals and animal models of obesity [63,64,65]. Epidemiology studies showed that decreased plasma adiponectin levels are inversely correlated with the risk of colon cancer [66,67]. Adiponectin knockout (APNKO) mice exhibited more tumor numbers and areas in dextran sodium sulfate (DSS)  and  1,2-dimethylhydrazine (DMH) induced colon cancer model through increasing the differentiation from epithelial cells to goblet cells and inhibiting goblet cell apoptosis. It indicated that adiponectin protected against chronic inflammation-induced colon cancer [68]. High-fat diet treated mice had more and larger colorectal tumors than chow-diet mice. Adiponectin administration decreased tumor growth through inhibiting angiogenesis [69,70]. In vitro experiments, adiponectin inhibits colon cancer cell growth in adiponectin receptor (AdipoR1- and -R2) positive HCT116, HT29, and LoVo cells through the AMP-activated protein kinase (AMPK)/mammalian target of rapamycin (mTOR) signaling pathway [71,72]. Moon et al. demonstrated that adiponectin directly regulated cell proliferation, migration, adhesion, and colon formation through regulation of metabolism, inflammation, and cell cycle in MCA38, HT29, HCT116, and LoVo cells [69]. These results indicate the potential inhibitory effect of adiponectin on the development of CRC. Together, leptin and adiponectin generally show opposite molecular effects on obesity and cellular behaviors. They are relevant but reverse players in obesity-related CRC.

#### 3.2.3. Estrogens

It is well established that estrogen contributes to obesity-associated hormone-responsive cancers, especially breast cancer [73,74]. The role of estrogen in obesity-associated CRC is complicated. First, estrogens have been found to reduce the risk of CRC [75]. Hormone replacement therapy confers protection against CRC, especially for lean women, as indicated by epidemiological data [76]. Estrogen replacement therapy in postmenopausal women reduces CRC-related mortality [77]. These cohort studies indicated estrogens may play a protective role in the pathogenesis of CRC. Interestingly, adipose tissues are also partial source of estrogen in addition to ovaries. Plasma estrogen levels are increased in obese men and postmenopausal women, because adipose tissue aromatase transforms androgenic precursors to estrogens [78]. However, several studies have shown that high BMI increased the risk of CRC in men and premenopausal women, but not postmenopausal women [79,80]. Adiposity also positively correlates with blood insulin, leading to increased IGF-1. The inducible effect of insulin/IGF-1 axis on CRC appears to be compromised by estrogen released from adiposity in postmenopausal women. In premenopausal women, the primary source of estrogen is ovary compared to adiposity. Thus, more hormone supplement cannot provide more benefits [79,81]. This concept has been suggested by several cohort studies showing a positive correlation between BMI and CRC risk in younger women (<55-year-old) but not in older women [79,80,82]. This association was further confirmed by the study subjected between BMI and CRC risk in premenopausal and postmenopausal women. The risk of CRC in postmenopausal women is independent of BMI [83]. Although the relationship among hormones, obesity, and CRC is not fully understood, these observations and reasonable speculation emphasize the same importance of weight control in both genders.

The effect of estrogen is mediated by its receptors, estrogen receptor (ER)-α and ER-β. The expression of ER-α is very low in normal colorectal tissues. However, the ER-α expression is increased with the development of colon cancer, and it positively correlates with CRC stages and worse survival [75]. ER-β is enriched in colon tissues [84]. The expression of ER-β is lower in colon tumor tissues compared with normal tissues and inversely correlates with the progression of CRC [85,86]. ER-β overexpression induced cell-cycle arrest and inhibited cell proliferation and tumor growth in SW480 cells and mouse xenografts model [87]. In the Apc^Min/+^ mouse model, estrogen treatment protected against CRC and increased the ratio of ER-β to ER-α [88]. Ablation of ER-β in Apc^Min/+^ mice significantly increased tumor formation, and treatment with estrogen could not prevent this phenotype [89]. These results indicate that ER-β is responsible for the protective effect of estrogens on colon tumorigenesis.

**Figure 4:**
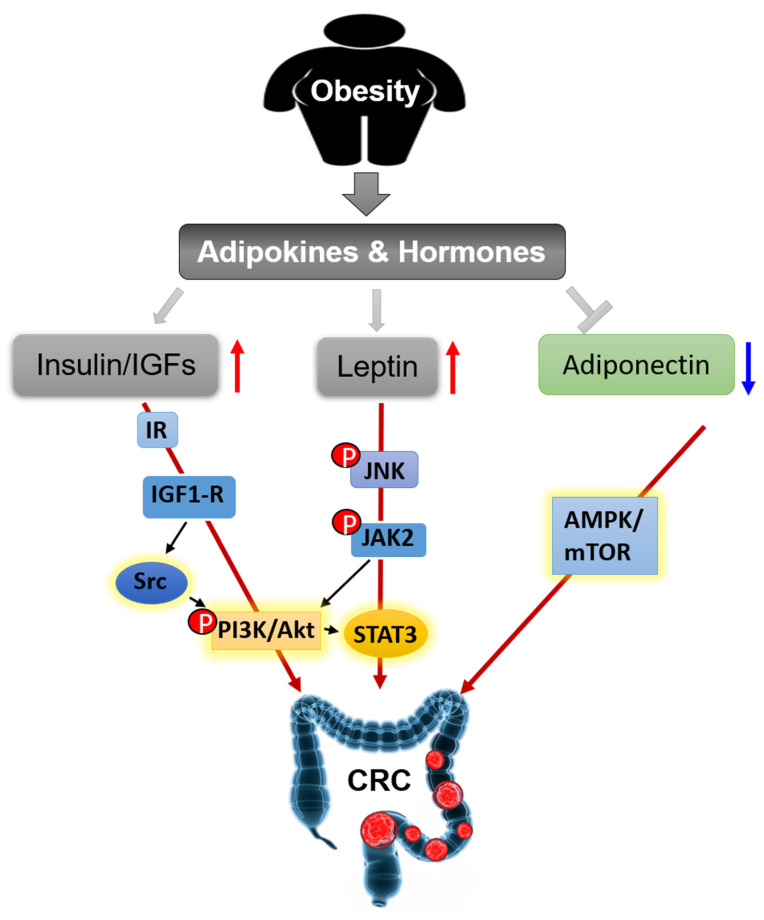
Obesity secreted adipokines and hormones contributing to pathogenesis of CRC.

We summarize the signaling pathways of obesity-secreted adipokines and hormones in the pathogenesis of CRC (Figure 4).

### 3.3. Inflammation

Obesity, as a characteristic of metabolic syndrome, is related to chronic low-grade inflammation in obese subjects, because of various pro- and anti-inflammatory cytokines produced by adipose tissues, including IL-6, TNF-α, CCL2, PAI-1, and others [90,91]. Chronic inflammation is a major link between obesity and tumor microenvironment in CRC.

Obesity is associated with circulating levels of IL-6. It has been reported that about 30% of circulating IL-6 was secreted from adipose tissues [91,92]. Circulating IL-6 is an important inflammatory factor in the acute inflammatory reaction which stimulates C-reactive protein (CRP) synthesis and secretion in the liver [91]. IL-6 is found in the tumor microenvironment of both murine and human colon cancer [93,94]. Prediagnostic plasma CRP, a general marker for inflammation, is also a reliable biomarker for CRC clinically [95]. Elevated levels of circulating CRP or IL-6 in CRC patients were associated with cancer progression, relapse, and worse survival [96,97]. IL-6 may act as a CRC-promoting cytokine due to its inflammatory property.

TNF-α is also secreted from adipose tissues. TNF-α expression in adipose tissues is positively correlated with the degree of obesity and associated type 2 diabetes mellitus(T2DM) [98,99]. The production of TNF-α is elevated in IBD patients and it is involved in the pathogenesis of IBD and associated CRC [100,101,102]. TNF-α can stimulate NF-κB activation, and the activation of IKK/NF-κB pathway is indispensable for colitis and colorectal carcinogenesis [103,104]. TNF-α promoted the proliferation and migration of CD44^+^CD133^+^ HT29 cells by activation of Wnt/β-catenin signaling pathway [101]. Treatment with low concentrations of TNF-α (20 µg/L) enhanced cell migration and invasion in HCT116 cells through upregulating tumor-associated calcium signal transduction protein 2 (TROP-2) by phosphorylation of extracellular signal-regulated kinase (ERK)1/2 signaling pathway [105].

CCL2, also known as monocyte chemoattractant protein 1 (MCP-1), is secreted by adipocytes and plays a crucial role in inflammatory reaction [106]. Circulating levels of pro-inflammatory CCL2 is also increased in obese subjects [107]. Tumor-associated macrophage induced inflammation is related to poor prognosis of CRC. CCL2 is an imperative monocyte-attracting chemokine stimulating the recruitment of macrophages into the sites of tumors [108]. Knockout CCL2 in Apc^Min/+^ mice (Apc^Min/+^/CCL2^−/−^) inhibited tumor growth and immune infiltration in colon cancer [109]. CCL2 facilitated the accumulation of polymorphonuclear myeloid-derived suppressor cells (PMN-MDSCs) into the tumor microenvironment and increased MDSC-mediated inhibition of T cells in a STAT3-dependent manner, and blocking CCL2 using antibodies reduced tumor growth and MDSC infiltration in a murine model of colitis-associated CRC [109]. Targeting inhibition of CCL2 may provide therapeutic benefits for the prevention and interception of CRC.

PAI-1 is encoded by *SERPINE1* gene in humans. It is secreted mainly by hepatocytes and endothelial cells, and partly by adipose tissues [110]. Clinically, PAI-1 expression in various tumors is higher than that in normal tissues [111]. The plasma PAI-1 level was increased in CRC patients, but it was not correlated with the risk of colonic carcinogenesis [112]. Recently, Gerard reported [113] that PAI-1 aggravated mucosal damage through PAI1–tPA axis and activation of transforming growth factor β (TGF-β) in human and murine colitis. Knockout PAI-1 or treatment with PAI-1 inhibitor reduced inflammation and mucosal damage in DSS- and *Citrobacter*-induced colitis [114]. PAI-1 is an important inducer of the inflammatory reaction in colonic epithelial cells.

**Figure 5:**
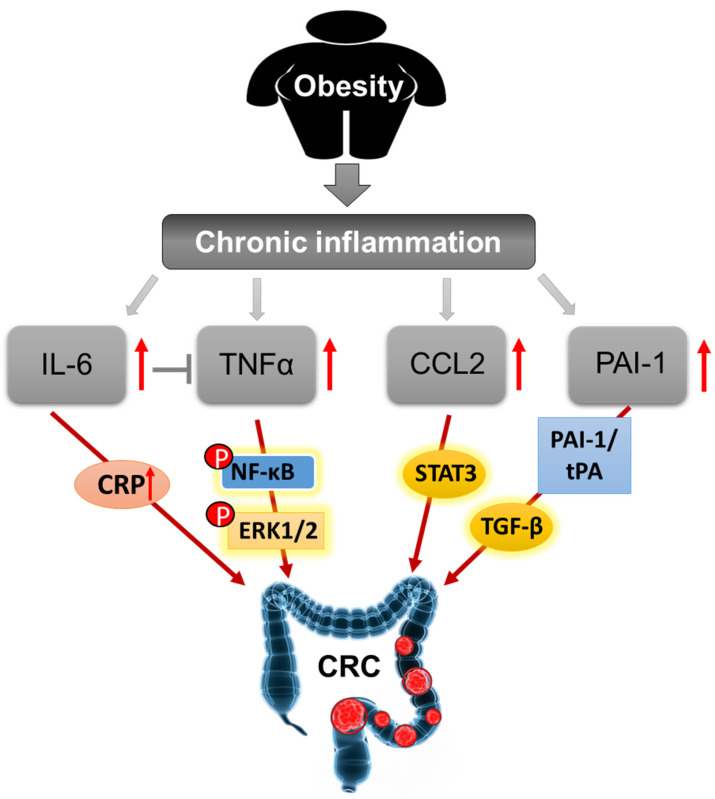
Schematic mechanisms of carcinogenesis of CRC induced by obesity-elicited chronic inflammation.

Low-grade chronic inflammation is a main feature of obesity, it mediates most of the obesity-related complications [115]. Inflammation also plays an important role in the tumor microenvironment of CRC to activate the signaling of proliferation, migration, and metastasis [116,117]. Therefore, obesity triggered chronic subclinical inflammation is a bridge linking obesity to colorectal carcinogenesis. We summarize the mechanisms by which obesity-induced chronic inflammation leads to the carcinogenesis of CRC (Figure 5).

### 3.4. Gut Microbiota

Gut microbiota has become increasingly important for health with the launch of the National Microbiome Initiative and Human Microbiome Project in America in recent years [118,119]. In humans, about 1.5 kg microbes reside in the gut and make up half of the fecal matter biomass [120]. Increasing evidence indicated that gut microbiota is considered as a potential factor in the pathogenesis of obesity and associated metabolic disorders, even cancers [121,122,123]. Understanding the role of gut microbiome in obese and CRC individuals will provide potential molecular insights and therapeutic targets to prevent or treat both diseases.

Germ-free animals are critical for studying the effect of microbes on host physiological and pathological processes. In different high fat and carbohydrate diets induced obesity models, germ-free animals have more food intake but gain less body weight compared with the conventional controls [122]. In carcinogen-induced and spontaneous colon cancer models, germ-free animals also show inhibited tumorigenesis in most cases [124]. Vannucci et al. reported that germ-free rats exhibited reduced tumor formation and enhanced anti-cancer immune response in AOM-induced CRC compared with conventional conditions [125]. T-cell receptor β chain and p53 double-knockout (TCRβ^−/−^, p53^−/−^) mice can spontaneously form colorectal tumors. The rate of tumor formation is about 70% in conventional mice. Whereas, there is almost no tumor in the germ-free mice [126]. Tomkovich et al. found that germ-free Apc^Min/+^ and IL10^−/−^ mice had less colorectal tumors compared to specific-pathogen-free and gnotobiotic controls, and polyketide synthase (pks)^+^ *Escherichia coli* promoted carcinogenesis mediated by colibactin [127].

Lipopolysaccharide (LPS) is an endotoxin produced by gram-negative bacteria in the gut and is associated with low-grade chronic inflammation [128]. Circulating LPS was elevated in high-fat diet (HFD) induced obesity due to a gut microbiome remodeling [129,130]. We recently found HFD increased the abundance of LPS-producing pathogens *Desulfovibrio* in mice [129]. Bacteria and endotoxins are prevented by intestinal mucosal barrier [131]. Increased intestinal permeability and systemic endotoxemia aggravated colitis and associated CRC. Bacteria secreted LPS directly exacerbates extracellular matrix adhesion and invasion in SW480, SW620, and CACO2 cells through activation of the urokinase plasminogen activator (u-PA) system in a TLR-4/NF-κB dependent manner [132]. Wenting et al. found that LPS increased the migration and invasion of colorectal cancer cells in vivo and in vitro by promoting epithelial-mesenchymal transition (EMT) and activation of SDF-1α/CXCR4/ NF-κB axis [133]. LPS participates in the enhancement of CRC malignant behaviors, and it may serve as a biomarker for CRC metastasis.

Gut microbiota can produce some beneficial metabolites, such as short-chained fatty acids (SCFAs). SCFAs are key mediators linking diet and gut microbiota to prevent obesity and related metabolic disorders [134,135]. SCFAs are the major source of energy for colonocytes, and important for gastrointestinal health to maintain intestinal barrier function [136]. SCFAs also play a beneficial role in CRC clinically [137]. Mechanically, SCFAs inhibited cell growth and differentiation, promoted cell-cycle arrest and apoptosis, and regulated histone acetylation to protect against CRC [138]. Given the potential benefits of SCFAs, they are also considered as useful probiotics to prevent CRC.

*Akkermansia muciniphila,* a genus of the phylum *Verrucomicrobia*, is a probiotic for preventing both obesity and CRC [139,140,141,142]. The Patrice group found *Akkermansia* protected against HFD-induced obesity through increasing intestinal endocannabinoids that reduced inflammation and enhanced gut barrier function [143]. Further, they found Amuc_1100, a specific membrane protein purified from *Akkermansia*, improved metabolic syndrome in obese and diabetic mice through TLR2 signaling [144]. In obese human volunteers, *Akkermansia* administration improved insulin sensitivity and inflammation, mildly reduced body weight, compared to placebo [145]. *Akkermansia* is also a crucial player in gastrointestinal disorders. Treatment with *Akkermansia* inhibited DSS-induced colitis in mice [146]. Amuc_1100 and pasteurized *Akkermansia* blunted colitis and associated CRC tumorigenesis through regulation of macrophages and CD8^+^ cytotoxic T lymphocytes in mouse colon [147]. *Lactobacillus casei* is a genus of *Lactobacillus*. Oral administration of *Lactobacillus casei* enhanced CD8^+^ T cell infiltration and inhibited colon carcinoma growth in tumor-bearing mice [148]. These data indicate that potential probiotic bacteria and beneficial metabolites are promising therapeutic agents for treating obesity and CRC.

### 3.5. Bile Acids

Bile acids (BAs), amphipathic molecules, mainly mediate intestinal dietary fat absorption. The primary BAs are synthesized from cholesterol in the liver and secreted into the intestine where pancreatic lipase is activated to form micelles and promotes nutrient absorption [149]. BAs also serve as signaling molecules to regulate farnesoid X receptor (FXR) and G protein-coupled receptor (GPCR) signaling, thereby maintaining energy and metabolic homeostasis [150]. BAs play key roles in lipid metabolism. The synthesis of BAs is associated with circulating triglyceride levels in patients with hyperlipoidemia [151]. Cholestyramine is a BA sequestrant commonly used for reducing high serum cholesterol levels in patients [152]. In the obesity models, total BAs were slightly increased, while conjugated BAs and deoxycholic acid (DCA) were dramatically elevated in plasma and liver [153,154]. The level of total BAs is correlated with BMI in obese patients [149]. BAs, especially secondary BAs, are potent carcinogens or promoters for CRC. Numerous studies reported that BAs are strong inducers for CRC tumorigenesis by damaging colonic epithelium, stimulating inflammatory reactions [155], inducing reactive oxygen species (ROS) production [156], promoting genomic instability, and resisting apoptosis [157]. Targeting BAs might be an effective strategy for the prevention and treatment of CRC.

FXR is a bile acid receptor (BAR), encoded by the *NR1H4* gene and highly expressed in the liver and intestine tissues [158]. FXR is a double-edged sword in obesity. Evans et al. reported activation of intestinal FXR by fexaramine inhibited obesity and increased adipose tissue browning through fibroblast growth factor 15 (FGF15) signaling [159,160]. On the other hand, Frank et al. found Glycine-β-muricholic acid (Gly-MCA), an intestine-specific FXR inhibitor, reduced obesity and associated metabolic dysfunction through inhibition of ceramide metabolism [161]. Additionally, FXR is a therapeutic target to protect against colorectal tumorigenesis. FXR inhibited colonic tumor growth in vivo. Knockout FXR in the Apc^Min/+^ mice promoted tumor progression and accelerated mortality through activation of Wnt/β-catenin signaling pathway [158,162]. T-β-MCA, a known FXR antagonist, was reported to promote CRC progression in HFD-induced APC^min/+^ mice through damaging DNA and increasing proliferation in leucine-rich repeat-containing G protein-coupled receptor 5 positive (LGR5^+^) cancer stem cells [163]. Given the anti-tumor activity, intestinal FXR has promising therapeutic value in treating CRC.

## 4. Conclusions

**Figure 6:**
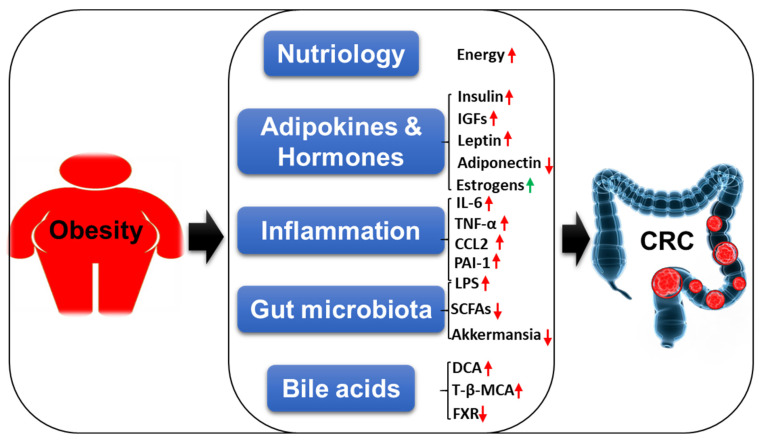
A schematic model of mechanistic insights linking obesity with CRC carcinogenesis. Red arrow indicates promotion, green arrow indicates protection.

Both obesity and CRC are global health burdens currently. Epidemiologic data indicate a positive correlation between obesity and CRC. Obesity plays a direct and independent role in colorectal carcinogenesis. In the present review, we described the epidemiology of obesity and CRC respectively, and then summarized the potential underlying mechanisms linking obesity to CRC in different aspects, including nutriology, adipokines and hormones, inflammation, gut microbiota, and bile acids as shown in Figure 6. In nurtiology, adipocytes in tumor microenvironment are an energy source for CRC growth. In adipose tissue secreted adipokines and inflammation, elevated levels of insulin, IGFs, leptin, and inflammatory cytokines (e.g., IL-6, TNF-α, CCL2, and PAI-1), and decreased levels of adiponectin in obese, which alone or together contribute to the formation and development of CRC. Interestingly, circulating estrogen level is increased in obese individuals. It is known that estrogen contributes to obesity related breast cancer, but the role of estrogen in obesity-associated CRC is controversial. Cohort studies showed that BMI affects males stronger than females in the carcinogenesis of CRC, indicating estrogen may have a protective effect in CRC. In gut microbiota, obesity induced gut microbiota dysbiosis increases harmful microbiota and metabolites (LPS) and decreases beneficial microbiota (*Akkermansia*) and metabolites (SCFAs), which might lead to CRC tumorigenesis. In bile acids, bile acids promote CRC progression, especially DCA and T-β-MCA which are increased in obesity. The carcinogenesis of CRC is promoted by the bile acid-dependent inhibition of FXR, which is a target for anti-CRC. Therefore, obesity induces complex biological activities to promote CRC tumorigenesis.

Besides obesity, epidemiologic evidence showed dietary and lifestyle factors include red/processed meat diet, low-fiber and high-fat diet, alcohol drinking, smoking, sedentary, and low physical activity are important environmental factors for CRC risk [164]. Genetic risk factors include familial adenomatous polyposis (FAP), and certain genetic mutations [165] (e.g., mutL homolog 1 (MLH1), adenomatous polyposis coli (APC), K-Ras (KRAS), and tumor protein p53 (TP53) genes). Environmental and genetic factors commonly contribute to CRC development. Reducing weight, improving diet, decreasing alcohol intake and smoking, and in addition to reducing sedentary time and increasing physical activity are likely to improve CRC incidence and mortality.

In summary, we mainly focus on the role of obesity in CRC. The potential underlying biological mechanisms linking obesity to CRC are warranted, although great strides have been made to understand the biological mechanisms in obesity and the pathogenesis of CRC, respectively. Obesity induces insulin, IGFs, leptin, IL-6, TNF-α, CCL2, and PAI-1, reduces adiponectin, and disturbs gut microbiota and bile acid homeostasis. These altered factors promote CRC carcinogenesis mediated by downstream signaling pathways. Our increased understanding of the link between obesity risk factors and CRC carcinogenic processes will help to uncover more promising therapeutic targets and approaches for obesity-related CRC treatment in the future.
